# Supply-demand balance in outward-directed networks and Kleiber's law

**DOI:** 10.1186/1742-4682-2-45

**Published:** 2005-11-10

**Authors:** Page R Painter

**Affiliations:** 1 Office of Environmental Health Hazard Assessment, California Environmental Protection, Agency, P.O. Box 4010, Sacramento CA 95812, USA

**Keywords:** nutrient supply networks, allometric scaling, metabolism

## Abstract

**Background:**

Recent theories have attempted to derive the value of the exponent α in the allometric formula for scaling of basal metabolic rate from the properties of distribution network models for arteries and capillaries. It has recently been stated that a basic theorem relating the sum of nutrient currents to the specific nutrient uptake rate, together with a relationship claimed to be required in order to match nutrient supply to nutrient demand in 3-dimensional outward-directed networks, leads to Kleiber's law (*b *= 3/4).

**Methods:**

The validity of the supply-demand matching principle and the assumptions required to prove the basic theorem are assessed. The supply-demand principle is evaluated by examining the supply term and the demand term in outward-directed lattice models of nutrient and water distribution systems and by applying the principle to fractal-like models of mammalian arterial systems.

**Results:**

Application of the supply-demand principle to bifurcating fractal-like networks that are outward-directed does not predict 3/4-power scaling, and evaluation of water distribution system models shows that the matching principle does not match supply to demand in such systems. Furthermore, proof of the basic theorem is shown to require that the covariance of nutrient uptake and current path length is 0, an assumption unlikely to be true in mammalian arterial systems.

**Conclusion:**

The supply-demand matching principle does not lead to a satisfactory explanation for the approximately 3/4-power scaling of mammalian basal metabolic rate.

## Introduction

Regression analyses of measurements of a physiological or structural variable *R *(*e.g*. cardiac output or pulmonary alveolar surface area) in mammals of different mass *M *have shown in many cases that the variable is closely approximated by a function of the form

*R = R*_1_*M*^*b*^,

which is often termed an allometric relationship [1,2]. A prominent example is Kleiber's law for scaling the basal metabolic rate, *B*, in mammals [3,4],

*B = B*_1_*M*^3/4^,

which is equivalent to scaling the specific basal metabolic rate, *B/M*, proportionally to *M*^-1/4^.

The search for a theory to explain Kleiber's law has recently focused on the nutrient distribution network formed by arteries and capillaries. Banavar *et al*. [5-7] argue that the law follows from basic properties of an outward-directed network (ODN). In the initial description of an ODN [5,6], Banavar, Maritan and Rinaldo (BMR) assume that a network consists of sites for nutrient uptake that are connected to a single source (*e.g*. the heart). An uptake site is located at each network branching point and at each terminal network point. Network distance *L*_*y *_along a path from the nutrient source *O *to a site *Y *is defined as the number of uptake sites on the path. The rate of uptake of nutrient at site *Y *is denoted *B*_*y*_. A network segment that goes from a site *X *to an adjacent site *Y *is termed the link *XY*, and the rate at which nutrient enters the link is termed the current and is denoted *I*_*xy*_. For a link that carries nutrient current from a site *X *to a site *Y*, the level of the link *XY *and the level of the site *Y *is defined as the network distance *L*_*y *_to the site *Y*. In an ODN, direction of flow is away from *O *on each link. The authors denote the sum of currents on all links *ΣI*_*xy*_, termed total network current, by *F*, which is shown to be defined by the equation

*F *= *ΣB*_*y*_*L*_*y*_.     (1)

The initial ODN theory is completed by the introduction of the relation:

*F *= *nE(B*_*y*_*)E(L*_*y*_*)*,     (2)

where *n *is the number of uptake sites and *E(B*_*y*_*) *and *E(L*_*y*_*) *denote average values.

In the first attempt to derive the law using Relation (2), total network current is assumed to be proportional to blood volume in the mammalian systemic arterial and capillary system, and this blood volume is assumed to be proportional to body mass. With the additional assumption that *E(L*_*y*_*) ∝ L*_*p*_, where *L*_*p *_is the linear dimension of the region supplied by the network, it follows that total uptake rate, *B*, scales as  and that blood volume and body mass scale as . Consequently, *B *scales as *M*^3/4^. However, body tissue density, *M/V*, is predicted in this model to scale as *L*_*p*_*= V*^1/3 ^[8,9]. If density were to scale as *L*_*p*_, the density of hippopotamus tissue would be more than ten times the density of mouse tissue and would far exceed the density of granite.

An additional problem in the BMR theory is that total network current, an abstract property of the arterial system, is not necessarily proportional to blood volume [10]. Furthermore, Relation (2) is not true in examples of ODNs where uptake occurs only at terminal sites [10].

In a second attempt to derive Kleiber's law using the concept of total network current, Banavar, Damuth, Maritan and Rinaldo (BDMR) add the assumption that networks are embedded in spatial regions "such that mass and volume scale isometrically" [7]. Cubic and square regions are examples of such isometric bodies. They also assume that body mass scales as body volume, , where *D *is the dimension of the region representing the body. Citing the previous attempt to derive Kleiber's law, they write

*F ∝ (L*_*p*_*/u)B*,     (3)

and claim that "Eq. 3 has been proven as a mathematical theorem" (*u *is the average physical distance between connected uptake sites). Next, they define the function

*r*_1_*= F/S*,     (4)

where *S *denotes the system's size (measured as area for a 2-dimensional system and volume for a 3-dimensional system). They define the "service volume"  by the relationship , and they consider the scaling of

*r*_2_*= l*_*s*_*/u*.

Clearly, *r*_2_, which is described as the rate "with which the metabolites are taken in at the level of the tissue," is defined by the relation

*r*_2_*∝ (B/S)*^-1/*D*^*/u*.     (5)

Next, BDMR state: "Maintaining a match between these two rates across body size would require that both rates scale with body mass in the same manner, *i.e*., if *r*_1 _∝  and *r*_2 _∝ , then *s*_1 _= *s*_2_. If this were not true, under changes of body mass either the supply of the metabolite would exceed the demand or vice versa." Based on this reasoning, they assert their supply-demand matching principle:

*r*_1_*∝ r*_2_.     (6)

Combining Relations (3) and (6) leads to Kleiber's law.

**Figure 1:**
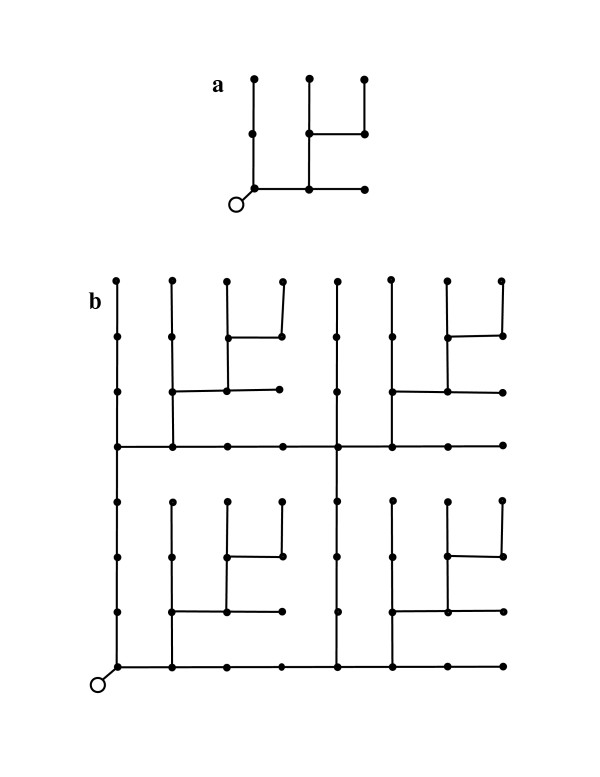
Simple lattice ODN models. a. A 3 × 3 ODN where current or nutrient is supplied by the link from the origin (large open circle) to the lower left corner. Uptake sites are denoted by closed circles. b. An 8 × 8 ODN where current or nutrient is supplied by the link from the origin (large open circle) to the lower left corner. Uptake sites are denoted by closed circles. Note that there are four identical embedded 3 × 3 outward-directed networks (*e.g*. the network in the upper right corner) within the 8 × 8 network.

BDMR state that their "conclusions are based on general arguments incorporating the minimum of biological detail and should therefore apply to the widest range of organisms" [7]. They support their theory with two lattice models of isometric ODNs, a 2 × 2 lattice and a 3 × 3 lattice. In their examples, the uptake rates are identical and the physical link lengths are identical throughout a lattice. Lattices with these properties are termed simple lattice networks. Figure 1a illustrates a simple 3 × 3 lattice ODN. Figure 1b illustrates an 8 × 8 simple lattice ODN with four embedded 3 × 3 lattice ODNs. The lattice in Figure 1a and the embedded lattices in Figure 1b are formally equivalent to the example provided by BDMR in their Figure 1b[7]. BDMR do not test their model using ODNs that are not square simple lattices.

## Results

While BDMR repeatedly state that they proved Relation (3) in their original publication on ODNs [7,11], they could not have proved this result. This can be demonstrated by considering ODNs in non-isometric solid bodies. (The networks considered in their original publication were not assumed to be isometric.) Consider two lattice ODNs that have identical spacing *u *between adjacent uptake sites and identical uptake rate *B*_*y *_at each uptake site. These two networks differ in their total network current (denoted, respectively, by *F*_1 _and *F*_2_), in their linear dimension (denoted *L*_*p*1 _and *L*_*p*2_) and in their number of uptake sites (denoted *n*_1 _and *n*_2_). If Relation (3) is correct, we can write

*F*_1_*/F*_2_*= (L*_*p*1_*n*_1_*)/(L*_*p*2_*n*_2_*)*.

However, this equation is a false statement whenever *L*_*p*1_*/L*_*p*2 _is an irrational number because both *F*_1_*/F*_2 _and *n*_1_*/n*_2 _are rational numbers. For example, when network 1 is a 2 × 3 × 4 lattice and network 2 is a 3 × 4 × 5 lattice, *L*_*p*1_*/L*_*p*2 _is (5/2)^1/3 ^and the above equation must be false.

For the isometric networks considered by BDMR, the ratio *L*_*p*1_*/L*_*p*2 _is a rational number, and Relation (3) is correct for some, but not all, families of isometric ODNs. In the remainder of this section, the theory of BDMR is evaluated in three ways. The first is an evaluation of its predictions for an ODN that is not a simple lattice. The second is an evaluation of whether Relation (6) is correct for simple outward-directed current network models, and the third is the identification of mathematical conditions required for the validity of the critical mathematical relationships, Relations (2), (3) and (6).

**Figure 2:**
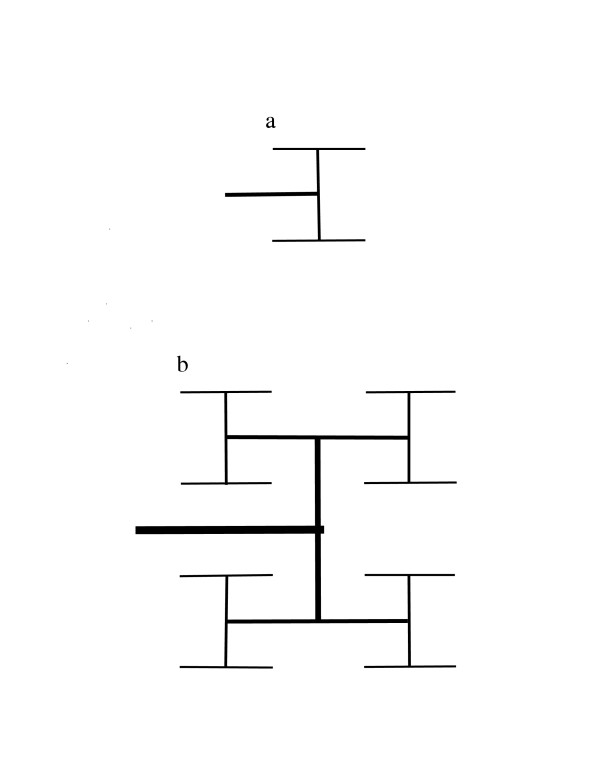
A 2-dimensional fractal-like, branching network model for an arterial tree. Blood enters the network through the structure represented as a thick horizontal line. Terminal arteries are represented by thin horizontal lines. a. A network that uniformly supplies a 2 × 2 area where the unit distance is the spacing between adjacent termini of small arteries. b. A network that uniformly supplies a 4 × 4 area.

**Figure 3:**
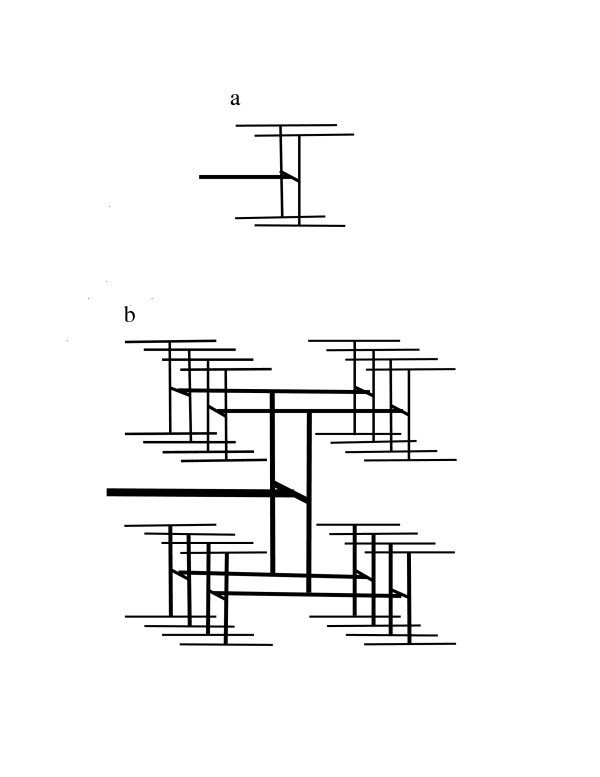
A 3-dimensional fractal-like, branching network model for an arterial tree. Blood enters the network through the structure represented as a thick horizontal line. Terminal arteries are represented by thin horizontal lines. a. A network that uniformly supplies a 2 × 2 × 2 volume where the unit distance is the spacing between adjacent termini of small arteries. b. A network that uniformly supplies a 4 × 4 × 4 volume.

If the logic used by BDMR to derive Relation (3) is correct for all ODNs that supply isometric regions, the assumption of Relation (6) should lead to the conclusion that Kleiber's law holds for outward-directed models of the arterial system that differ from the lattice models presented by BDMR. To see if this is correct, we apply this assumption to the well-known outward-branching "fractal-like" model studied by West *et al*. [12]. Figure 2 and Figure 3 illustrate how an outward-bifurcating network can be folded inside a square or cube with side or edge length equal to *2*^*i*^*l*_*t*_, where *i *is a positive integer and *l*_*t *_is the linear dimension of the region supplied by terminal uptake sites. The supply network for a square starts with an H-shaped network of linear dimension *L*_*p*_*/2 *that is connected to the nutrient source (Figure 2a). The network is extended by iteratively connecting each terminal site to an H-shaped structure that is one-half the size (in terms of linear dimension) of the structures added in the previous step (Figure 2b). For a network that supplies a cube, we start with two parallel H-shaped structures of linear dimension *L*_*p*_*/2 *that are connected by a conduit of length *L*_*p*_/2. This structure, termed an H-H structure, is illustrated in Figure 3a. This network is extended by iterative additions of H-H structures of one-half the dimension of the previously added H-H structure (Figure 3b). Each added structure is connected at its midpoint to a terminus. Iterative addition of smaller and smaller H-shaped structures gives the fractal lung model of Mandelbrot [13], and iterative addition of H-H structures gives a 3-dimensional fractal model. An infinite sequence of additions gives an area-filling network of fractal dimension 2 for the 2-dimensional network and a space-filling network of fractal dimension 3 for the 3-dimensional network. These networks have the topological structure of a Cayley tree. Consequently, the claim [6] that Cayley-tree networks are not plausible models of the mammalian arterial network because a Cayley tree "for large enough size, cannot exist in any finite-dimensional space" is incorrect.

Because these outward-bifurcating networks are folded inside a square or a cube, their scaling behavior can be directly compared with the scaling behavior of isometric lattices embedded in regions of identical shape and size. The networks shown in Figure 2 and Figure 3 start with a single link and bifurcate at each branch point until a terminal uptake site is reached at path length (number of links) *k*. The uptake rates at terminal sites and branch points are denoted *B*_*a *_and *B*_*b*_, respectively. The number of terminal uptake sites *2*^*k*-1 ^is equal to *(L*_*p*/ _*l*_*t*_*)*^*D*^, where *L*_*p *_is the length of the side or edge. Total network uptake *B *is *B*_*a*_*2*^*k*-1^*+ B*_*b*_*[2*^*k*-1 ^- 1*]*, and total network current *F *is *B*_*a*_*k2*^*k*-1^*+ B*_*b*_*[(k-2)2*^*k*-1 ^- 1*]*.

First, we assume that *B*_*b *_is negligible compared to *B*_*a*_. The biological justification for this simplification is that *B*_*b *_represents nutrient uptake by endothelial cells in arteries and by smooth muscle cells in small arteries and arterioles, and this uptake may be very small compared to the nutrient uptake from capillaries represented by *B*_*a*_. Application of the scaling assumption in Relation [6] to the formulas for *B *and *F *in this example gives the relation *(B/S)*^-1/*D*^*/u ∝ (B/S)k*, which is equivalent to *B/S ∝ (uk)*^-*D*/(*D*+1)^. This expression would be equivalent to Kleiber's law if *k *scaled as *S*^1/*D*^. However, for the networks in Figure 2 and Figure 3, the length of a link between neighboring sites is a constant (denoted u), and *k *scales as *Dln(S*^1/*D*^*/u)/ln(2)+1*. If it is assumed that *B*_*b *_= *B*_*a*_, the approximations *2*^*k*-1 ^≈ *2*^*k*-1 ^- 1 and *(k-2)2*^*k*-1 ^≈ *(k-2)2*^*k*-1 ^- 1 lead to the relationship *B/S ∝ [u(k-1)]*^-*D*/(*D*+1^, which is again very different from Kleiber's law.

While the above example shows that Kleiber's law cannot be derived from general properties of ODNs using Relation (6), the possibility remains that the derivation of BDMR is correct for outward-directed lattices and that the arterial system is more accurately modeled as a simple lattice ODN (where Relation (3) is true) than as a Cayley tree. If this is true, the validity of a claim that Kleiber's law is correct for lattice-like arterial supply-demand models depends on the validity of the supply-demand matching principle in Relation (6).

To see if this principle is correct in general for lattice ODNs that supply metabolites, we consider an example where a lattice network of pipes supplies liquid nutrient to nearly identical mature animals (*e.g. *inbred adult laboratory rats). A single animal is located in a cage at each vertex and at each terminal site. The length of a link connecting neighboring sites is a constant (denoted u), and each animal takes up nutrient through a valve that provides liquid to the animal only when it sucks and swallows all the liquid provided. Uptake by a caged animal is measured as the amount of nutrient or water ingested at the site per day and is denoted *B*_*y*_. This model is analyzed because the overall uptake rate is determined by demand, as is nutrient uptake in the "Allometric Cascade" model for basal metabolic rate scaling [14,15]. In such a biological example, supply is exactly matched to demand, and the logic used by BDMR to justify Relation (6), if correct, should predict the scaling of the system in the following cases: In case 1, the number of uptake sites, *n*, of the lattice is increased while *u *and *B*_*y *_remain constant. In case 2, *u *is increased while *n *and *B*_*y *_remain constant. In case 3, *B*_*y *_increases while *n *and *u *remain constant. Case 3 can be achieved by replacing adult animals in a nutrient-supply lattice with young growing animals that increase their uptake rate as they grow. These three cases are easily translated into equivalent examples where the lattice supplies electrical power to residences located at each lattice junction.

The scaling behavior of *r*_1 _and *r*_2 _in these three examples is listed in Table 1 along with the scaling relations for each of these supply-demand lattices. In each case, the network maintains a match between supply and demand. In none of the three cases does the network do this by "maintaining a match" between the rates *r*_1 _and *r*_2 _"across body size." Furthermore, none of these cases has the scaling of Kleiber's law. In case 1 and case 2, where the size of the system is increased, *r*_2 _is clearly an intensive property of the system while *r*_1 _depends on system size. In case 3, supply is matched to demand by balancing an increase in *r*_1 _with a decrease in *r*_2_. Total network current and *r*_1 _increase directly in proportion to *B*_*y*_. On the other hand, *r*_2 _decreases in proportion to . Clearly, *r*_2 _is not the rate of "the demand for delivered metabolites" which increases in proportion to *B*_*y*_, nor are the units of *r*_2 _"inverse time units" as claimed by BDMR.

**Table 1:** Scaling of *r*_1 _and *r*_2 _in three cases of parameter variation in supply-demand lattice ODNs with uptake determined by demand.

Parameter variation	Scaling of:		Scaling of B
		
	*r*_1_	*r*_2_	
*n *↑	∝ *S*^1/*D*^	∝ *S*^0^	∝ *S*
*u *↑	∝ *S*^-1^	∝ *S*^0^	∝ *S*^0^
*B*_*y *_↑	∝ *By*	∝ *By*^-1/*D*^	∝ *B*_*y*_

Another peculiarity of the ODN theory becomes apparent when it is applied to ODN lattices embedded in a larger ODN lattice. Figure 1b illustrates four 3 × 3 lattices supplied at a corner and embedded in an 8 × 8 lattice supplied at a corner. If Relationship (6) is correct for the ODNs in the figure, then the metabolic rate for each 3 × 3 lattice, denoted *B*_3_, should be *a(3*^2^*)*^2/3^, where *a *denotes the constant of proportionality. Similarly, the metabolic rate of the 8 × 8 lattice, denoted *B*_8_, is *a(8*^2^*)*^2/3^. From conservation of energy, it is clear that 4B_3 _must be less than or equal to B_8_, *i. e., 4(3*^2^*)*^2/3 ^must be less than or equal to *(8*^2^*)*^2/3^. However, 4(3^2^)^2/3 ^is an irrational number between 17 and 18, while (8^2^)^2/3 ^is equal to 16. A similar argument applied to eight 4 × 4 × 4 cubic lattices embedded in a 10 × 10 × 10 cubic lattice leads to a contradiction if it is assumed that the same 3/4-power scaling relationship applies to the entire lattice and to the embedded lattices. This argument can be generalized to show that an allometric scaling law, *B = B*_1_*S*^*α *^for isometric lattices with invariant *u *cannot have an exponent less than 1.

The above network examples show that the relations derived by BDMR are not true for all ODNs with supply-demand balance. We now identify assumptions that are not stated by BDMR but that guarantee the validity of these relations. First we define the conditions required for the validity of Relation (2). To do this, we apply the following basic theorem from statistical theory: For random variables *X *and *Y*, the well-known formula for *E(XY)*, the average value of the product of random variables, is

*E(XY) = E(X)E(Y) + Covariance(X,Y)*.

Application of this theorem to Relation (1) gives

*F *= *nE(B*_*y*_*)E(L*_*y*_*)+ nCovariance(B*_*y*_, *L*_*y*_*)*

which simplifies to

*F *= *E(L*_*y*_*)B + nCovariance(B*_*y*_, *L*_*y*_*)*

Therefore, Relation (2) is correct for an ODN if and only if *Covariance(B*_*y*_, *L*_*y*_*) *is 0, and the covariance is 0 if *B*_*y *_and *L*_*y *_are independent. If *B*_*y *_is invariant, independence is assured.

Now assume that Relation (2) is true for an ODN. We denote the physical length of a network link from site *X *to site *Y *and carrying current toward site *Y *by *u*_*xy*_. Next, define a path to a site *Y *as a sequence of connected links carrying outward-directed current from the source to site *Y*. The physical length of this path is the sum of the lengths of the links that form the path. To derive Relation (3), we assume that all paths to a site *Y *have the same path length (denoted *d*_*y*_) and that the length of all links carrying current to site *Y *is the same (denoted *u*_*y*_). These assumptions are true for simple lattice ODNs and for fractal-like ODNs. The number of paths that pass through or terminate at site *Y *is denoted by ν_*y*_. We define the average path length as *E(d*_*y*_*) *and assume that *E(d*_*y*_*) *is proportional to *L*_*p*_. In the sum that defines the numerator of *E(d*_*y*_), the sum of the values of *u*_*y *_is *ν*_*y*_*u*_*y*_. Therefore,

*E(d*_*x*_*) = (Σν*_*y*_*u*_*y*_*)/n*

which is equivalent to

*E(d*_*y*_*) = E(ν*_*y*_*)E(u*_*y*_*) + Covariance(ν*_*y*_, *u*_*y*_*)*

In computing *E(ν*_*y*_*)*, we note that the sum of the values of *ν*_*y *_for all level *1 *sites is equal to the number of level *1 *links on all paths. In general, the sum of the values of *ν*_*y *_for all level *j *sites is the number of level *j *links on all paths. Therefore, *E(ν*_*y*_*) *is the sum of all links on all paths divided by the number of paths, *i.e*. *E(ν*_*y*_*) = E(L*_*y*_*)*. Consequently,

*E(d*_*y*_*)/u = E(L*_*y*_*) + Covariance(ν*_*y*_, *u*_*y*_*)/u*

which shows that, when *L*_*p*_*∝ E(d*_*y*_*) *and the two additional assumptions on physical path length are true, Relation (3) follows from Relation (2) if and only if *Covariance(ν*_*y*_, *u*_*y*_*)/u *is *0 *or is proportional to *E(L*_*p*_). In isometric lattice models with constant spacing between uptake sites, this covariance is *0*, and *E(d*_*y*_*)/u *and *E(L*_*y*_*) *are proportional to *E(L*_*y*_). However, in the models of Figure 2 and Figure 3, this covariance is not *0 *because both *ν*_*y *_and *u*_*y *_decrease from level *1 *links to level *k *links. Furthermore, for these bifurcating ODNs, *E(L*_*y*_*) *is approximately proportional to the logarithm of *E(L*_*y*_*) *[8]. Consequently, Relation (3) is not true for these ODNs.

Finally, we show that when Relation (3) is true for an isometric 3-dimensional ODN, assuming that Relation (6) is true is equivalent to assuming that Kleiber's law is true. If total current in a family of networks is described by Relation (3), if mass and volume scale proportionally to  and if metabolic rate is described by Kleiber's law, then *B*^-4/3^*∝ M*^-1^, and *B*^-1/3^*∝ B/M*. Multiplying both sides by *L*_*p*_*/u *gives , and substituting *F *for *(Lp/u)B *gives Relation (6). The steps of this argument can be reversed to show that if Relation (3) and Relation (6) are true, then Kleiber's law is true. Therefore, for isometric networks where Relation (3) is true, the supply-demand principle in Relation (6) and Kleiber's law are equivalent statements.

## Discussion and conclusion

The incorrect prediction of the BMR model that body tissue density scales as *L*_*p *_is not a prediction of the BDMR model, which contains the assumption that body mass scales as body volume. However, the related current model of Dreyer and Puzio does predict that the mass of blood in a body scales as *L*_*p *_[16,17].

One issue in evaluating the model of BDMR is the validity of Relation (2) and Relation (3). BDMR state that they proved these relations as theorems [7,11]. However, a counterexample to their "theorem" of Relation (2) has been published [10], and the above results show that when uptake rates are not independent of path length, there is no reason to believe that Relation (2) or Relation (3) is true.

A second issue in evaluating the model of BDMR is whether a network of cubic lattices or the bifurcating ODN model of West *et al*. [12] more closely resembles the mammalian system of arteries and capillaries. This is a critical question because the basic assumptions of BDMR, Relation (2) and Relation (3), are true for simple lattices but not for the bifurcating ODNs of Figure 2 and Figure 3. The arterial system is clearly more similar to the West *et al*. model than to a simple lattice [18].

The principle claimed to be required to match supply to demand in ODNs is not correct for plausible conceptual models where supply must be matched to demand, and it does not lead to Kleiber's law for "fractal-like" ODNs. Therefore, the supply-demand matching principle does not lead to a satisfactory explanation for the approximately 3/4-power scaling of mammalian basal metabolic rate.

The supply-demand principle of BDMR has also been investigated by Makarieva *et al*. [19]. They, too, conclude that *r*_2 _is not the rate of "the demand for delivered metabolites" which increases in proportion to *B*_*y*_, nor are the units of *r*_2 _"inverse time units" as claimed by BDMR.

A final issue in the evaluation of the model of BDMR and other models that predict 3/4-power scaling of the basal metabolic rate is that experimental support for Kleiber's Law is rapidly eroding. As reviewed by Heusner [19] and Dodds *et al*. [8], the slope of the allometric scaling expression is less than 3/4. Furthermore, these investigators showed that the slope for mammals weighing less than 10 kg is approximately 2/3 while the slope for mammals weighing more than 10 kg is approximately 3/4. More recently, White and Seymour [21] showed that, following a correction for the effect of body temperature on metabolic rate, the slope is 0.67 for a very large collection of data (619 mammalian species). Statistical analysis of these data yields a slope that is less than 2/3 for animals smaller than 1 kg and a slope greater than 3/4 for animals larger than 50 kg [22].

The erosion of support for Kleiber's law should not result in a loss of interest in explanations for the scaling of metabolic rate. To the contrary, large collections of metabolic data that exhibit upward curvature support models based on physiological and anatomical considerations [14,15,22] but do not support Kleiber's law. Such models may focus attention on relationships at the heart of metabolic scaling issues, the physiological relationships between tissue blood flow and tissue metabolic rate.
